# Our Friend Bryden

**Published:** 1895-08

**Authors:** 


### Our Friend Bryden.

The death of Dr. W. Bryden, which is chronicled in this month's Journal, removes a familiar figure from the walks of veterinary life. His presence will be missed at the meetings of the United States Veterinary Medical Association, where he was wont to be. From its inception to the present day his interest was untiring, though in the past two years clouded by the inroads of disease that impaired his mental vigor and acute reasoning power. He was a man of original thought and genius, and lived for many years in the future, in the scope of his perceptions and reasonings, as they applied to diseases and defects of the locomotor apparatus. Charitable, with all in the broadest acceptance of the word, his loss will be mourned by every one who knew him in the noonday of his vigor and mental power.

